# P04.59. National surveys show lower well-being among yogis yet efficacy trials show favorable results: does dose-response resolve the contradiction?

**DOI:** 10.1186/1472-6882-12-S1-P329

**Published:** 2012-06-12

**Authors:** C Park, K Riley, M Stewart, E Bedesin, T Braun

**Affiliations:** 1 University of Connecticut, Storrs, USA; 2 Kripalu Center for Yoga & Health, Stockbridge, USA

## Purpose

General population studies indicate yoga practitioners have poorer mental and physical health than non-practitioners. However, yoga interventions have demonstrated improved mental and physical wellbeing. To date, researchers have not addressed this contradiction. We tested a dose-response relationship, hypothesizing that those who do more yoga would be better off than those who do less.

## Methods

Yoga practitioners across the US (82 men, 456 women, mean age 44 years) completed standardized questionnaires on the Internet, including full time teachers (N=44) and part time teachers (N=118). Demographics were similar to those of nationally representative samples of yoga practitioners. Dose was calculated as minutes/week of studio, home, and studio + home practice along with studio + home minutes/week x years of practice.

## Results

Consistent with previous research, our sample was in poorer emotional and physical health than the general population (moderate Depression and severe anxiety and stress scores on the DASS-21, and slightly poorer physical health scores on the SF-12 physical health subscale (PCS; 47.33 vs. 50). However, yoga dose and wellbeing were positively correlated. For the whole sample, PCS was positively related to studio and studio+home doses and DASS-21 stress scores were negatively correlated with studio+home dose and studio+home dose x years practiced. Spiritual well-being and relaxation skills were positively correlated with dose, as well. Similar but somewhat different patterns were found in sample subsets (e.g., those who were also yoga teachers, practitioners of different types of yoga).

## Conclusion

Similar to previous research, we found that yoga users had lower well-being than the general population. Individuals with more distress and ill health may turn to yoga for relief (Birdee et al, 2008). However, among those who do practice yoga, more practice is associated with better well-being, supporting our dose-response hypothesis. Longitudinal research is necessary to examine causal links between yoga dose and wellbeing.

